# Impact of depressive symptoms on medication adherence in older adults with chronic neurological diseases

**DOI:** 10.1186/s12888-024-05585-7

**Published:** 2024-02-16

**Authors:** Aline Schönenberg, Konstantin G. Heimrich, Tino Prell

**Affiliations:** 1 Department of Geriatrics, Halle University Hospital, Halle (Saale), Germany; 2 Department of Neurology, Jena University Hospital, Jena, Germany

**Keywords:** Older adults, Medication adherence, Depression, Network analysis, Beck depression inventory

## Abstract

**Background:**

Nonadherence to medication contributes substantially to worse health outcomes. Especially among older adults with chronic illness, multimorbidity leads to complex medication regimes and high nonadherence rates. In previous research, depressive symptomology has been identified as a major contributor to nonadherence, and some authors hypothesize a link via motivational deficits and low self-efficacy. However, the exact mechanisms linking depressive symptomology and nonadherence are not yet understood. This is in part because the often-employed sum scores cannot do justice to the complexity of depressive symptomology; instead, it is recommended to assess the influence of individual symptoms.

**Methods:**

Following this symptom-based approach, we performed correlation, network and regression analysis using depressive symptoms as depicted by the items of the revised Beck Depression Inventory II (BDI) to assess their influence with nonadherence in *N* = 731 older adults with chronic neurological diseases. Nonadherence was measured with the self-report Stendal Adherence to Medication Score (SAMS).

**Results:**

Even when controlling for sociodemographic and health-related covariates, the BDI remained the most influential contributor to nonadherence. Across different methods, Loss of Interest and Difficulty with Concentration were identified as particularly influential for nonadherence, linking nonadherence with other affective or somatic BDI items, respectively. Additionally, Fatigue, Problems with Decision Making, Suicidal Thoughts, and Worthlessness contribute to nonadherence.

**Conclusion:**

Using a symptom-driven approach, we aimed to understand which depressive symptoms contribute to higher levels of nonadherence. Our results refine previous hypotheses about motivation and control beliefs by suggesting that it is not merely a lack of beliefs in the efficacy of medication that connects depressive symptoms and nonadherence, but rather an overall lack of interest in improving one’s health due to feelings of worthlessness and suicidal tendencies. This lack of interest is further substantiated by already sparse resources caused by changes in concentration and fatigue. In order to improve health outcomes and reduce nonadherence, these associations between depressive symptoms must be further understood and targeted in tailored interventions.

**Supplementary Information:**

The online version contains supplementary material available at 10.1186/s12888-024-05585-7.

## Background

With advancing age, the prevalence of chronic diseases in general and neurological diseases in particular increases. The World Health Organization (WHO) estimates that more than 20% of adults aged 60 years have a mental or neurological disease; other projections predict that the number of these older adults will double by 2050, leading to an increasing burden of age-related diseases worldwide [1, 2].

Importantly, most chronic conditions are treated with medication. According to the American Center for Disease Control and Prevention, in 2016, 85% of US citizens aged 60 or older received a prescription for medication [3]. Because older adults often have multiple conditions, they have to adhere to complex medication regimens [2, 4, 5]. To achieve optimal health outcomes, it is essential that patients take these medications as prescribed. Nonadherence describes a situation where patients do not take their medicines as agreed on with their healthcare providers [4, 6]. Nonadherence rates remain high, with a recent review estimating nonadherence at 43% [7]. As nonadherence reduces the effectiveness of medication and/or can lead to adverse health events due to side effects or inappropriate drug interactions [8], nonadherence is generally associated with poorer health outcomes and quality of life (QoL) [9, 10].

The reasons for nonadherence are multifaceted; in a review, Yap and colleagues summarize five overall domains of adherence barriers: medication factors such as medication complexity and frequency of change, physician factors such as communication and satisfaction, system factors including finances and availability, miscellaneous factors, and patient factors such as age and gender, cognition, personality, and overall health [11]. Among these, depressive symptoms as patient factors have been identified as particularly detrimental [12, 13]. This association between depressive symptoms and nonadherence is particularly harmful because poorer health and depressive symptoms are interrelated, leading to a downward spiral of poorer physical and mental health. As poorer health in old age is also associated with more medical prescriptions, the association between nonadherence and depressive symptoms makes the latter an ideal starting point for improving nonadherence rates. Thus, across many different studies, including different patient groups and measurement tools, depressive symptoms have been consistently identified as influential [11, 14, 15].

Of note, depression is a highly heterogeneous construct that includes both affective symptoms, such as loss of interest, hopelessness, sadness and lack of pleasure, and somatic symptoms concerning sleep, appetite and concentration [16–19]. Additionally, much like nonadherence, depressive symptoms are complex and may differ in their manifestation between individuals [20–23]. Because of this complexity, new efforts have been made to expand the view of depressive symptomology towards a symptom-based approach. Accordingly, researchers are proposing to move away from the traditional idea of depression being a single (latent) construct that causes its corresponding symptoms, and instead to focus on these very symptoms as a self-sustaining, interactive system [21, 23–25]. This approach suggests that symptoms influence and trigger each other in cyclic relationships that cannot be satisfactorily accounted for by summarizing depression in a single diagnostic criterion or total score. This symptom-based approach is based on research demonstrating a) significant associations between depressive symptoms, b) symptom overlap between depression and other psychiatric disorders, and c) the overall lack of a replicable (factor) structure of depression as an overall diagnostic term across individuals [20–23, 25–28]. This symptom-based approach not only recognizes the complexity of depressive symptoms, but also allows a better understanding of which of the many depressive symptoms have an impact on, for example, health, QoL, or adherence [29].

Despite the close association between nonadherence and depressive symptoms, it is not well understood how exactly depressive symptoms exert their influence. While several studies report an effect of higher depression sum score values on higher levels of nonadherence [11–13], for example a meta-analysis by Grenard et al. estimates an odds ratio of 1.76 for nonadherence in patients with depression compared to patients without depression [15], these studies cannot explain which aspects of depressive symptoms deliver this effect. Many authors hypothesize about potential effects of reduced concentration or motivation as a connecting factor [14, 15, 30]; however, the symptom-driven approach described above may shed light on which depressive symptoms contribute primarily to nonadherence. While this approach has been applied to depressive symptoms in other contexts [31–35], to the best of our knowledge, it has not yet been done to assess its relation with nonadherence. Therefore, we applied different methods to assess the relationship between nonadherence and individual depressive symptoms to understand by which mechanisms depressive symptomology is linked to nonadherence.

## Methods

### Study design, setting and participants

The data used for this secondary analysis were taken from the NeuroGerAd study, an observational study on medication adherence and related psychosocial factors conducted on the wards of Neurology at Jena University Hospital, Germany, from 2019 to 2020. Detailed information on the study design and collected data can be found in the published study materials [36–38]. Briefly, older patients with common neurological main diagnoses as confirmed by the hospital’s leading physicians received a comprehensive assessment during their hospital stay. Initial study inclusion criteria comprised age (≥ 60 years, or ≥ 55 years with multi-morbidity), cognition (no severe cognitive impairments as indicated by Montreal Cognitive Assessment > 18 or diagnosis of dementia, no delirium), and absence of severe depression. Of the original 910 participants included in the study, *N* = 731 completed both the dependent and independent variable of interest for this manuscript, and were thus included in the present analysis.

### Variables

We extracted the following variables for the present analysis:

- The dependent variable was depressive symptomology as assessed with the revised Beck Depression Inventory (BDI) [39, 40]. The BDI encompasses 21 items assessing the presence and intensity of different depressive symptoms on a 4-point Likert scale.
- The key independent variable was medication adherence measured with the Stendal Adherence to Medication Score (SAMS), a self-report scale encompassing a sum score as well as the sub-scales *Modification* of medication, *Forgetting* to take medication, and *Missing Knowledge* about medication. Each of the 18 items is posed as a 4-point Likert scale ranging from 0 to 4, with higher scores indicating higher levels of nonadherence. The SAMS has undergone testing across a range of patient groups, such as neurological patients, chronic pain patients, and patients who have received kidney transplants, and the three sub-scales have been replicated in various studies [41–45]. In our study, we calculated the sub-scales as the mean of the respective items (Cronbach’s α: *Total Score* = 0.83 [95% CI 0.82-0.85], *Forgetting* = 0.73 [95% CI 0.70-0.76], *Modification* = 0.84 [95% CI 0.82-0.86], and *Missing Knowledge* = 0.79 [95% CI 0.77-0.81]). As no universally accepted cut-off point for nonadherence is defined, we treated the SAMS as a continuous variable [46].

In addition, to evaluate the relative contribution of the BDI, we included the following covariates:

- Age (years), Sex (Male/Female), Living Situation (Alone/not Alone), Marital State (Married or in a relationship/not Married), Education (low ≤ 8 years, medium 9 – 11 years, high ≥ 12 years corresponding to the German education system)
- Type of medical main diagnosis as given by physicians during the patients’ hospital stay (Movement Disorder, Cerebrovascular Disorder, Neuromuscular Disorder, Epilepsy, Miscellaneous Disorders) and number of different medications taken daily
- Self-Rated Health (SRH) according to item 1 of the SF-36. This item asks patients to rate their general health (“in general, would you say your health is…?”) on a scale of 1 = excellent to 5 = poor [47, 48].
- Satisfaction with healthcare indicated by Healthcare Climate Questionnaire (HCCQ) The HCCQ utilizes 15 Likert-Scale items to assess patients’ perception of support for autonomy, competence, communication, and empathetic support. It is summarized as a mean score, with higher scores indicating a higher overall satisfaction with the provided care. It has been tested and validated in previous studies [49–51].
- Cognition assessed with the Montreal Cognitive Assessment (MoCA). The MoCA is one of the most commonly used cognitive screenings with high sensitivity especially for differentiation between unimpaired cognition and mild cognitive impairment. It incorporates not only memory and orientation but also abstraction, language/fluency, and visuospatial tasks. A maximum of 30 points can be received, with higher scores reflecting better performance. In addition to utilizing the overall sum score as a continuous variable, different cutoffs are proposed in various patient populations [52–55].
- As we included not only patients with movement disorders but generally older adults, we measured Mobility as indicated by the Timed Up and Go (TuG) test. During the TuG, patients are asked to stand up from a chair, walk a set distance, turn around and re-take their seat, assessing overall mobility required for every-day tasks [56]. The TuG is a validated and reliable measure for mobility also in impaired populations [57, 58].
- Personality according to the Big Five Inventory-10 (BFI) [59]. The BFI is the most commonly used and validated questionnaire to assess personality based on the Big Five theory including the traits openness, neuroticism, agreeableness, conscientiousness, and extraversion. The BFI-10 has five subscales with two Likert-scale items for each of the traits. Scale scores are then calculated as the participant’s mean response. Its validity has been confirmed previously in extensive German samples [60].

### Statistical methods

We used descriptive statistics (Mean and SD or Median and IQR) to describe the included patients. Using linear regression, we initially confirmed the association between BDI and SAMS while controlling for covariates. Subsequently, we performed network analysis (NA) [61–63] using the R-package *bootnet* [61] as an exploratory tool to map out the relation between the SAMS sum score and sub-scales, and depressive symptoms represented by the BDI items. Unlike traditional modelling approaches, NA does not assume an underlying latent factor to account for links between variables, but rather assumes that the included variables influence each other in a cyclic relationship. Especially for psychosocial items, this approach is beneficial as it assumes that items, e.g. symptoms in a questionnaire such as the BDI, are interrelated and assesses their interplay rather than reducing a phenomenon as complex as depressive symptomology down to one latent factor [21, 24–26].

This approach has recently been employed to study the complexity of mental health disorders, especially depression [22, 31, 32, 35, 61, 64]. The Gaussian Graphical Model (GGM) based on polychloric correlation for ordinal variables maps the relationships between two variables while controlling for all other variables in the network [65]. Consequently, the network plot does not contain mere correlations; two items can be strongly correlated but unconnected in the network if their association is delivered via other variables. Thus, NA can help understand the potential flow of information between different variables [66]. Of note, NA is an exploratory tool that we mainly used to visualize the complex interactions between the BDI items and the SAMS, allowing for the assessment of interconnection between items rather than reducing the data down into (orthogonal) factors or single latent constructs [66]. Although centrality measures exist to assess the influence of particular items within the network, we intentionally do not report them, mainly because centrality indices only indicate the importance of items relative to all items in the network, but not relative to specific constructs such as the SAMS. Thus, centrality indices do not provide useful information for our specific purpose [61, 67, 68].

Visually, NA displays two components: the variables (BDI items and SAMS scores), called *nodes*, and their connecting *edges.* Edges display the strength of the association with their thickness and the direction with their color, with red edges depicting negative associations. present edge indicates that, when conditioning on all other inter-item relationships in the network, a relation between two items remains. In contrast, the absence of an edge between two nodes indicates independence of those two nodes after conditioning on all other nodes. The nodes are then depicted graphically using the Fruchterman-Reingold algorithm, placing the nodes within the network based on the strengths of their associations. This means that nodes with strong connections are positioned in close proximity [69].

In NA with multiple variables, all edges are drawn per default, leading to a network that is difficult to interpret. Therefore, we used the Extended Bayesian Information Criterion with Graphical Least Absolute Shrinkage and Selection Operator (EBICgLasso) to shrink the absolute weights of the correlations towards zero, effectively reducing the number of edges to produce a sparse network [61, 70]. The hyper-parameter was set to 0.5. The stability of NA can be assessed using a case-dropping nonparametric bootstrap: if the correlation stability coefficient (CS-C) remains above 0.5, a proportion of the study sample can be dropped without major changes in the NA properties [61].

Lastly, we used linear regression with elastic net regularization to assess the contribution of the different BDI items on the SAMS variance. When using the BDI items as regressors, we performed Elastic Net Regularization with tenfold cross-validation to detect the optimal alpha and lambda combination [71, 72]. Elastic Net is a penalty-based combination of Ridge and Lasso regression to perform variable selection and prevent overfitting. This makes elastic net a beneficial approach when a multitude of independent variables is included in a model, when these variables are correlated, and/or sample sizes are small [71, 72]. The variables identified as relevant based on a reduction of the mean squared error (MSE) in the elastic net can then be entered into a final linear model. All elastic net models were compared to regular linear regression models with all included variables using the *performance-*package to detect the best-fitting model. Elastic Net was performed with the *glmnet* package in R [71].

Assumptions for linear regression were assessed with the *performance*-package in R [73]. All analyses were performed in R Version 4.3.1. [74]. P-values below 0.05 denote statistical significance, 95% confidence intervals (CIs) are given where possible. All visualizations were computed using *ggplot2* [75] or *qgraph* for the NA [69].

## Results

The included 731 patients had a mean age of 70.2 years (SD ± 8.61), ranging from 55 to 96 years. Of these, 326 patients (44.6%) were female (see Tables 1 and 2 for a descriptive overview).

**Table 1:** Sociodemographic information on included patients

Variable	Mean (SD)	Median (IQR)	N
Age	70.2 (8.61)	70 (14)	731
	**N**	**%**	**N**
Gender: female	326	44.6	731
Education			724
Low	224	30.9	
Medium	249	34.4	
High	251	34.7	
Marital Status: married	496	68.7	722
Living Situation: not alone	527	75.4	699
Diagnosis			731
Movement Disorder	237	32.4	
Cerebrovascular	191	26.1	
Neuromuscular	143	19.6	
Epilepsy	35	4.8	
Miscellaneous	125	17.1	

*IQR* interquartile range, *SD* standard deviation

**Table 2:** Descriptive statistics of included variables

Variable	Mean (SD)	Median (IQR)	N
BDI	9.68 (7.51)	8 (9)	731
SAMS	6.16 (7.59)	4 (8)	731
Modification	0.219 (.53)	0 (0.17)	731
Missing Knowledge	.446 (.79)	0 (0.75)	731
Forgetting	.418 (.57)	0.25 (0.50)	731
MoCA	23.50 (2.71)	23 (4)	731
HCCQ	5.62 (1.13)	5.9 (1.3)	692
TuG	10.60 (4.48)	10 (4)	477
Number of Drugs	5.74 (3.68)	5 (5)	697
	**N**	**%**	**N**
BFI			701
Neurotic	81	11.8	
Open	114	16.3	
Extroverted	148	21.1	
Conscientious	298	42.5	
Agreeable	60	8.7	
SRH (SF-36 Item 1)			721
1—Excellent	4	0.6	
2 – Very Good	18	2.5	
3 – Good	209	29.0	
4 – Fair	356	49.4	
5 – Poor	134	18.6	

*BDI* Beck Depression Inventory II, *BFI* Big Five Inventory, *HCCQ* Healthcare Climate Questionnaire, *MoCA* Montreal Cognitive Assessment, *SAMS* Stendal Adherence to Medication Scale, *SRH* Self-Rated Health, *TuG* Timed Up and Go

As a first step, we confirmed the association between the BDI and the SAMS that we reported in previous manuscripts as a basis for subsequent analyses (Supplement Table 1A).

In a univariate linear regression model (F(1, 729) = 52.23, *p* < 0.001, adjusted *R*^2^ = 0.07), the BDI was significantly associated with the SAMS sum score (est = 0.26, *p* < 0.001, 95% CI [0.19; 0.33]).

When adding the covariates to the model (F(23, 386) = 5.62, *p*. < 0.001, adjusted *R*^2^ = 0.201), the BDI remained a significant predictor of the SAMS (est = 0.36, *p* < 0.001, 95% CI [0.26; 0.45]) along with sex, HCCQ, and SRH (see Supplement Table 1B, Model 1). The BDI was also identified as a significant contributor to all the SAMS sub-scales even with adjustment for covariates (Supplement Table 1B, Models 2–4). Having confirmed the general relationship between SAMS and BDI, we aimed to understand which aspects of depressive symptomology as described by the BDI items deliver this influence. For this purpose, we performed subsequent analyses using the BDI items.

**Fig. 1:**
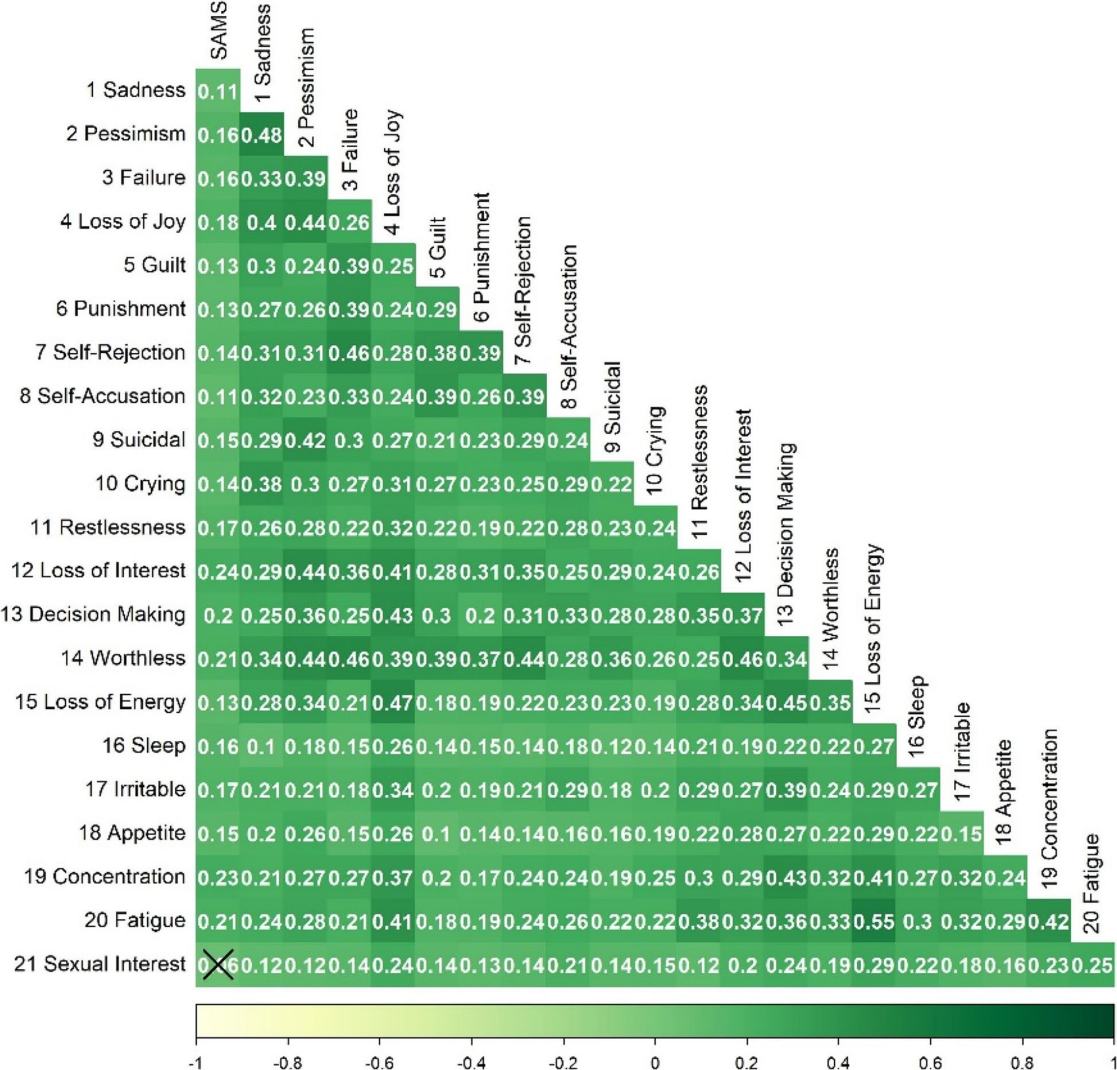
Spearman correlation for SAMS and BDI-II Items based on significance level of .05. Note: Values give correlation coefficients. Crossed-out values did not reach significance

Spearman correlations between the BDI items and the SAMS in the sum score (Fig. 1) and in the SAMS sub-scales (Supplement Figs. 1–3) were low to moderate but statistically significant for most items. However, for the Missing Knowledge sub-scale, only items 1–3 (Sadness, Pessimism and Failure), 10–14 (Crying, Restlessness, Loss of Interest, Decision Making, Worthlessness), and 18–19 (Appetite, Concentration) reached statistical significance. *Forgetting* was significantly associated with all items except for items 7 (Self-Rejection), 10 (Crying), 16 (Sleep) and 21 (Sexual Interest). The *Modification* scale was significantly correlated with all BDI items. The SAMS sum score is most strongly correlated with item 12 (Loss of Interest), 19 (Problems with Concentration), 20 (Fatigue), 14 (Worthlessness), and 13 (Decision Making). Looking at the SAMS sub-scales, the *Forgetting* sub-scale showed highest correlation with BDI items 12 (Loss of Interest), 19 (Concentration), 20 (Fatigue) and 13 (Decision Making) as well, although with lower loadings than for the SAMS sum score. For the *Missing Knowledge* sub-scale, items 19, 14 and 12 still showed highest associations, as well as item 18, and for the *Modification* sub-scale again items 12, 13, 14, and 20 as well as item 17 (Irritability) showed highest correlations.

**Fig. 2:**
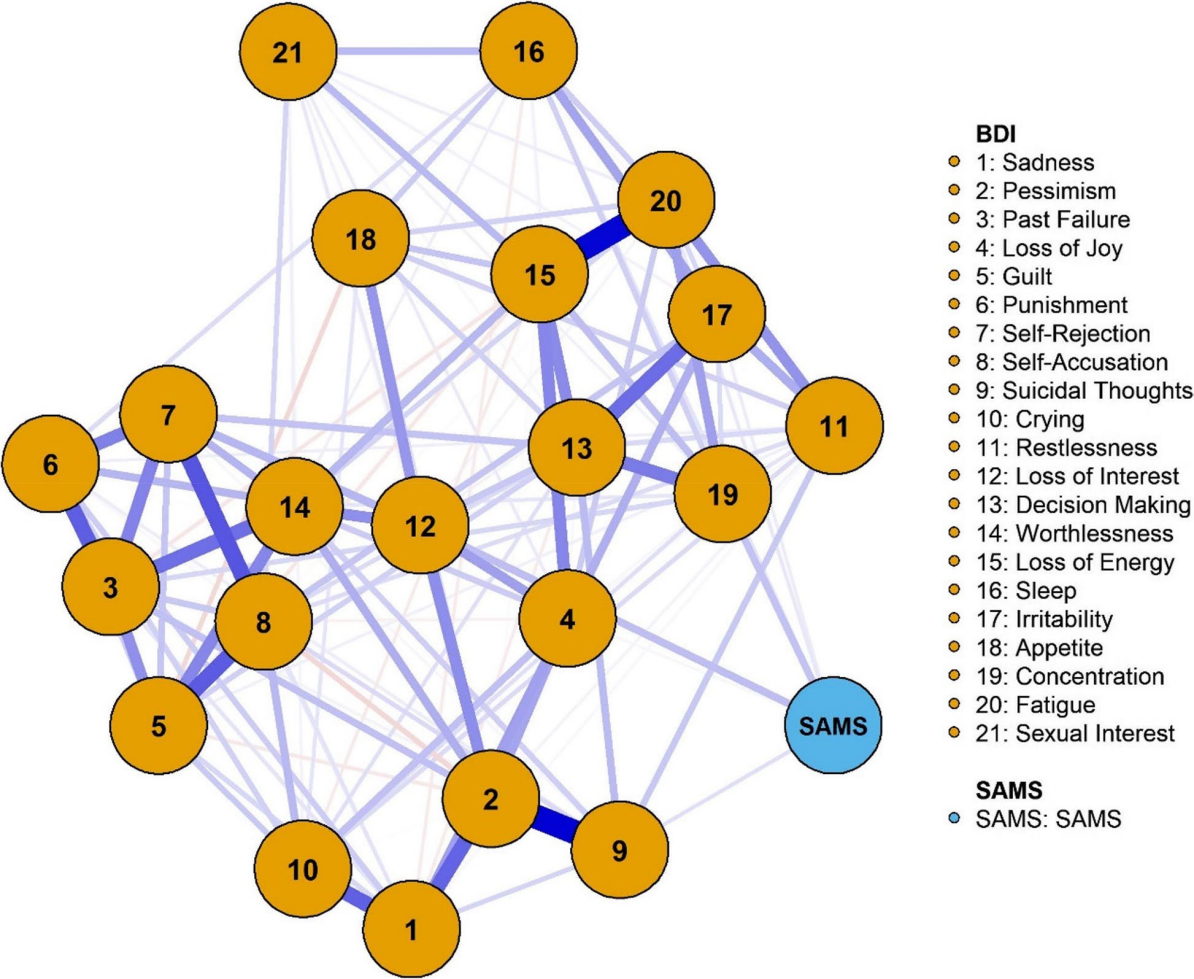
Network Analysis of SAMS Sum Score and BDI items

Correspondingly, in the Network for the total SAMS (Fig. 2), direct connections were present between the SAMS sum score and items 19 (Concentration) and 12 (Loss of Interest). Additionally, the SAMS was directly connected to BDI-II item 9 (Suicidal Thoughts), and weakly with items 17 (Irritability) and 20 (Fatigue). Visually, item 19 appears to connect the SAMS with other somatic BDI-items, while item 12 serves as a gateway to other affective BDI symptoms. Case-dropping bootstrap revealed the network to be sufficiently stable with a CS-C of 0.595, displaying 132/231 possible edges. Having established an overall relationship between the SAMS sum score and the BDI, we then used the SAMS sub-scales to provide more refined information about how the various BDI symptoms are related to aspects of nonadherence. (Supplement Figs. 4–6). All networks revealed CS-Cs above the required 0.5.

The Network for the *Forgetting* sub-scale confirmed direct associations between forgetting and items 12 (Loss of Interest), 9 (Suicidal Thoughts), and 20 (Fatigue), as well as weakly with item 19 (Concentration). Again, items 12 and 9 connect nonadherence with other affective BDI items, while items 19 and 20 link *Forgetting* with the somatic symptoms. The network for *Missing Knowledge* generally showed weaker relations, direct connections for knowledge were present with item 15 (Loss of Energy) as a connection to somatic BDI symptoms, and items 12, 14 (Worthlessness), and 3 (Failure). In the network for *Modification*, this sub-scale was directly connected to somatic symptoms via items 16 (Sleep) and 20, as well as weakly with 15 and 21 (Sexual interest). Weaker direct links were present with affective symptoms via items 12, 10 (Crying) and 8 (Self-Accusation).

Because NA is primarily an exploratory approach, we used linear regression with elastic net regularization to identify the BDI items most relevant in explaining SAMS variance. Regression analysis for the SAMS sum score and sub-scales yielded similar results as the NA. Accordingly, for the SAMS sum score, the BDI items 12 (Loss of Interest) and 19 (Concentration) were identified as main contributors to SAMS variance (Table 3).

**Table 3:** Linear Regression with Elastic Net Regularization for SAMS sum score with all BDI items as predictors

*Predictors*	**SAMS**		
	*Estimates*	*CI*	*p*
(Intercept)	3.56	2.61 – 4.51	**< 0.001**
bdi 3	1.01	-0.17 – 2.18	0.093
bdi 5	0.41	-1.07 – 1.90	0.584
bdi 11	0.61	-0.23 – 1.45	0.154
bdi 12	1.56	0.54 – 2.58	**0.003**
bdi 14	0.11	-1.11 – 1.33	0.858
bdi 16	0.35	-0.26 – 0.96	0.259
bdi 19	1.07	0.16 – 1.97	**0.021**
bdi 20	0.44	-0.49 – 1.36	0.354

*BDI* Beck Depression Inventory, *CI* confidence interval, *Sams* Stendal Adherence to Medication Scale

N 730, R^2^ / R^2^ adjusted 0.091 / 0.081

F(8, 721) = 9.02, *p* < .001

When looking at the *Forgetting* sub-scale (see Supplement Table 2, Model 1), only item 12 significantly contributed to explained variance of the sub-scale, while items 12, 15 and 19 contribute significantly to the *Missing Knowledge* (Supplement Table 2, Model 2) subscale. Finally, *Modification* (Supplement Table 2, Model 3) was related only to item 12.

## Discussion

Depressive symptomology has previously been identified as closely related to nonadherence, both in our data and in other studies [11, 14, 15, 36]. However, new approaches suggest that depressive symptomology needs to be considered at the symptom level rather than using sum scores [21, 24–26]. Therefore, we set out to examine the impact of depressive symptoms, as measured by the BDI, on medication nonadherence using correlation, network and regression analysis.

Overall, our data confirm that depressive symptoms and nonadherence are closely related, with the BDI sum score alone explaining 7% of SAMS variance. When socio-demographic and health-related covariates were included, the BDI still retained the strongest explanatory value in the model. Therefore, we used item-level correlation, network and regression analyses to explore this relationship between the BDI and SAMS in depth.

Both for the SAMS sum score and the sub-scales, BDI item 12 (Loss of Interest) has been identified as influential across all methods. The associations between depressive symptoms and adherence vary slightly depending on which sub-scale, i.e. which type of nonadherence, is considered, but overall items 12 and 19 (Problems with Concentration) were found to be directly related to nonadherence. Additionally, items 20 (Fatigue), 14 (Worthlessness) and 13 (Problems with Decision Making) were identified to contribute to nonadherence. In the NA, item 9 (Suicidal Thoughts) also showed direct associations with nonadherence for SAMS sum score and *Forgetting*.

Although there is no replicable structure of the BDI due to the high complexity and individuality [76], the BDI is often thought to incorporate both cognitive-affective and somatic symptoms [39, 77, 78]. Generally, higher levels of nonadherence as measured by the SAMS sum score were associated with other affective symptoms via Loss of Interest (Item 12) and with other somatic symptoms via Concentration (Item 19) and Fatigue (Item 20), indicating a multi-component association between depressive symptoms and nonadherence.

The effect of cognitive problems such as lack of concentration and unintentional forgetting of medication has been reported in previous studies [11, 14, 15, 79]; our data again indicate that not taking medication may be associated with concentration deficits as well as with a general physical weakness. Of note, the *Forgetting* sub-scale representing unintentional nonadherence was associated with both Concentration and Fatigue, as well as with a lack of interest and a feeling of worthlessness. The *Modification* sub-scale was primarily related to loss of interest, indicating a general carelessness about the correctness of medication intake. In the NA, also items 8 and 10 (Crying and Self-Accusation) were linked with higher levels of *Modification*; however, for this sub-scale the somatic symptoms appear to be more influential. Thus, the NA shows links with Fatigue (item 20) and Sleep Problems (item 16), which together with the influence of item 12 point towards a general lack of care and interest in one’s medication. This is in line with the association found between nonadherence and item 9 (Suicidal Thoughts), as well as item 14 (Worthlessness) that has been reported in the NA and regression for SAMS sum score as well as *Knowledge* and *Forgetting* sub-scales. These associations suggest an underlying general belief that taking care of one’s health is not worth the effort. Our results indicate overall that patients with higher levels of depressive symptomology may care less about their own well-being and survival due to general feelings of worthlessness and loss of interest in their well-being; and accordingly do not invest in their own health, especially when cognitive and energy resources are already scarce.

In their review, Grenard and Colleagues propose a “lack of energy, motivation, […], feelings of hopelessness and changes in cognition […]” [15] as pathways linking depressive symptoms with nonadherence. Our results confirm this hypothesis. Similarly, Goldstein and colleagues even suggest psychological counseling using motivational interviewing as a means to improve medication nonadherence [30], pointing to the importance of motivation and control beliefs in illness. Similarly, Schüz et al. identified the beliefs in efficacy and necessity of medication as predictors of nonadherence [80, 81], suggesting that the beliefs in the ability and necessity to improve one’s health are essential for adherence [41, 82]. In contrast, self-efficacy and locus of control are often reduced in persons with higher levels of depressive symptoms [83–85], and depressive symptoms have been shown to influence expectations and interpretations of health in older adults [86]. These results indicate an association between depressive symptomology and nonadherence via lack of beliefs in the ability to influence health; our present result substantiate these findings with the addition of worthlessness and loss of interest, suggesting that it is not only a lack of self-efficacy and control but also a lack of willingness to devote resources to the improvement of one’s own health in particular due to not feeling worthy. Additionally, our results highlight that these resources may also be scarce in the first place due to lack of concentration and problems with fatigue and sleep.

According to NA, this overall lack of interest (item 12) seems to bundle the other affective symptoms to culminate in nonadherence, while concentration (item 19) bundles somatic symptoms. Although with cross-sectional data, it is not possible to assess whether other affective symptoms result in lack of interest or whether lack of interest causes the other symptoms. While NA differs from traditional modelling by allowing the co-presence of connections and plotting the potential flow of information rather than taking into account the individual contribution of each variable separately, it remains an exploratory analysis especially when using cross-sectional data. Longitudinal analyses using symptom-based approaches such as NA that include more fine-tuned data such as motivation, self-efficacy and control perceptions as covariates may provide a more detailed understanding of the association between nonadherence and the broad bandwidth of depressive symptomology.

### Limitations

Our study is not free of limitations. Firstly, the single-center study design and specific study population hinders generalizability, although we did choose this particular cohort of older adults with neurological chronic diseases due to its high relevance and predisposition for depressive symptoms [2]. Although NA can provide useful insight into the structure of data, it requires large datasets in order to be sufficiently stable; thus subgroup-analyses concerning very specific patient populations, age groups or gender differences are not always feasible. Additionally, cross-sectional data cannot indicate causality, thus the analyses should be repeated with longitudinal data in different settings. Furthermore, both the depressive symptoms and nonadherence questionnaires are based on self-report; although self-reported measurements carry a risk of bias, they offer an opportunity to evaluate various forms of nonadherence and their underlying causes, which cannot be achieved through the use of more objective measures [46, 87]. Furthermore, when using valid scores, self-reports can provide reliable information on nonadherence behavior. Of note, the patients included in our study did not receive a psychiatric assessment, thus the depressive symptoms reported in our data are not indicative of Major Depressive Disorders. While the use of a questionnaire such as the BDI is useful as it provides an assessment of various different symptoms, it would be beneficial to repeat these analyses with patients at different intensities of depressive symptomology after professional psychiatric assessment.

## Conclusion

Modern research approaches highlight the need to assess depressive symptomology on symptom level to do justice to its high complexity. Based on this approach, we utilized several methods to assess the association between depressive symptoms and nonadherence to medication. Our results are in line with previous hypotheses suggesting a lack of cognitive resources and motivation or control beliefs. Additionally, they refine these hypotheses by highlighting that it is not merely a lack of beliefs in the efficacy of medication that connects depressive symptoms and nonadherence, but rather an overall lack of interest in improving one’s health due to feelings of worthlessness and suicidal ideas. This lack of interest is further substantiated by already sparse resources caused by changes in concentration and fatigue on the other hand.

### Supplementary Information

**Additional file 1:** **Supplement Table 1.** Linear Regression for SAMS and SAMS sub-scales using the BDI (A) and Covariates (B). **Supplement Table 2.** Linear Regression after Elastic Net Regularization for SAMS sub-scales using BDI items as predictors. **Supplement Figure 1.** Spearman Correlations for SAMS Forgetting and BDI Items. **Supplement Figure 2.** Spearman Correlations for SAMS Missing Knowledge and BDI Items. **Supplement Figure 3.** Spearman Correlations for SAMS Modification and BDI Items. **Supplement Figure 4.** Network for SAMS Forgetting and BDI Items. **Supplement Figure 5.** Network for SAMS Missing Knowledge and BDI Items. **Supplement Figure 6.** Network for SAMS Modification and BDI Items.

## Data Availability

An anonymized version of the dataset used in this analysis is available from: Prell, T., & Schönenberg, A. (2022). Data on medication adherence in adults with neurological disorders: The NeuroGerAd study. OSF. 10.17605/OSF.IO/KUAPH.

## Abbreviations

BDI: Beck Depression Inventory II
BFI: Big Five Inventory
CI: Confidence Interval
CS-C: Correlation Stability Coefficient
EBICgLasso: Extended Bayesian Information Criterion with Graphical Least Absolute Shrinkage and Selection Operator
GGM: Gaussian Graphical Model
HCCQ: Healthcare Climate Questionnaire
IQR: Interquartile Range
M: Mean
MoCA: Montreal Cognitive Assessment
MSE: Mean Squared Error
NA: Network Analysis
SAMS: Stendal Adherence to Medication Score
SD: Standard Deviation
SRH: Self-Rated Health
TuG: Timed Up and Go
