# Supplementary material for: Impact of depressive symptoms on medication adherence in older adults with chronic neurological diseases
Source: BMC Psychiatry. 2024 Feb 16;24:131. doi: 10.1186/s12888-024-05585-7 (PMC10870557; doi:10.1186/s12888-024-05585-7)
Supplement: Supplementary file 1 — Additional file 1: Supplement Table 1. Linear Regression for SAMS and SAMS sub-scales using the BDI (A) and Covariates (B). Supplement Table 2. Linear Regression after Elastic Net Regularization for SAMS sub-scales using BDI items as predictors. Supplement Figure 1. Spearman Correlations for SAMS Forgetting and BDI Items. Supplement Figure 2. Spearman Correlations for SAMS Missing Knowledge and BDI Items. Supplement Figure 3. Spearman Correlations for SAMS Modification and BDI Items. Supplement Figure 4. Network for SAMS Forgetting and BDI Items. Supplement Figure 5. Network for SAMS Missing Knowledge and BDI Items. Supplement Figure 6. Network for SAMS Modification and BDI Items. [file 12888_2024_5585_MOESM1_ESM.docx]

[**Supplement Table 1.** Linear Regression for SAMS and SAMS sub-scales using the BDI (A) and Covariates (B) 2](#_Toc157524774)

[**Supplement Table 2.** Linear Regression after Elastic Net Regularization for SAMS sub-scales using BDI items as predictors 3](#_Toc157524775)

[**Supplement Figure 1.** Spearman Correlations for SAMS Forgetting and BDI Items 4](#_Toc157524776)

[**Supplement Figure 2.** Spearman Correlations for SAMS Missing Knowledge and BDI Items 5](#_Toc157524777)

[**Supplement Figure 3.** Spearman Correlations for SAMS Modification and BDI Items 6](#_Toc157524778)

[**Supplement Figure 4.** Network for SAMS Forgetting and BDI Items 7](#_Toc157524779)

[**Supplement Figure 5.** Network for SAMS Missing Knowledge and BDI Items 8](#_Toc157524780)

[**Supplement Figure 6.** Network for SAMS Modification and BDI Items 9](#_Toc157524781)

## **Supplement Table 1.** Linear Regression for SAMS and SAMS sub-scales using the BDI (A) and Covariates (B)

| Predictors | **Est.** | **CI** | **p** | **Est.** | **CI** | **p** | **Est.** | **CI** | **p** | **Est.** | **CI** | **p** |
| --- | --- | --- | --- | --- | --- | --- | --- | --- | --- | --- | --- | --- |
| A Model with BDI Only | Model 1A: SAMS | | | Model 2A: Forgetting | | | Model 3A: Missing Knowledge | | | Model 4A: Modification | | |
| Intercept | 3.63 | 2.76 – 4.50 | **<0.001** | 0.28 | 0.21 – 0.34 | **<0.001** | 0.31 | 0.22-0.40 | **<0.001** | 0.08 | 0.01-0.14 | **0.014** |
| BDI | 0.26 | 0.19 – 0.33 | **<0.001** | 0.01 | 0.01 – 0.02 | **<0.001** | 0.01 | 0-01-0.02 | **<0.001** | 0.01 | 0.01-0.02 | **<0.001** |
| Model Fit | N = 731, R^2^/R^2^ adjusted = 0.067 / 0.066, F(1, 729) = 52.23, p < .001 | | | N = 731, R^2^/R^2^ adjusted 0.037 / 0.035, F(1, 729) = 27.87, p < .001 | | | N= 731, R^2^/R^2^ adjusted = 0.018 / 0.016, F(1, 729) = 13.12, p < .001 | | | N = 731, R^2^/R^2^ adjusted = 0.045 / 0.043, F(1, 729) = 34.12, p < .001 | | |
| B Model with Covariates | Model 1B: SAMS | | | Model 2B: Forgetting | | | Model 3B: Missing Knowledge | | | Model 4B: Modification | | |
|  | **Est.** | **CI** | **p** | **Est.** | **CI** | **p** | **Est.** | **CI** | **p** | **Est.** | **CI** | **p** |
| Intercept | 9.31 | -0.43 – 19.04 | 0.061 | -0.05 | -0.86 – 0.76 | 0.906 | 0.45 | -0.58 – 1.48 | 0.388 | 0.61 | -0.14 – 1.35 | 0.111 |
| BDI | 0.36 | 0.26 – 0.45 | **<0.001** | 0.01 | 0.00 – 0.02 | **0.009** | 0.02 | 0.01 – 0.03 | **<0.001** | 0.03 | 0.02 – 0.03 | **<0.001** |
| Age | 0.01 | -0.06 – 0.09 | 0.776 | 0.00 | -0.01 – 0.01 | 0.842 | 0.01 | 0.00 – 0.02 | **0.028** | -0.00 | -0.01 – 0.00 | 0.291 |
| Gender [male] | 1.85 | 0.65 – 3.05 | **0.003** | 0.13 | 0.03 – 0.23 | **0.013** | 0.15 | 0.02 – 0.28 | **0.021** | 0.07 | -0.03 – 0.16 | 0.159 |
| Education [medium] | -0.88 | -2.38 – 0.63 | 0.253 | 0.12 | -0.00 – 0.25 | 0.056 | -0.18 | -0.34 – -0.02 | **0.028** | -0.05 | -0.17 – 0.06 | 0.385 |
| Education [high] | -0.14 | -1.64 – 1.35 | 0.851 | 0.12 | 0.00 – 0.25 | **0.049** | -0.13 | -0.29 – 0.03 | 0.108 | 0.01 | -0.10 – 0.12 | 0.860 |
| Marital [married] | 1.55 | -0.48 – 3.58 | 0.134 | 0.14 | -0.03 – 0.31 | 0.098 | -0.05 | -0.27 – 0.16 | 0.625 | 0.16 | 0.00 – 0.31 | 0.046 |
| Living [not alone] | -0.41 | -2.62 – 1.80 | 0.715 | -0.03 | -0.21 – 0.16 | 0.762 | 0.08 | -0.16 – 0.31 | 0.521 | -0.10 | -0.27 – 0.07 | 0.247 |
| MoCA | -0.11 | -0.35 – 0.12 | 0.337 | 0.01 | -0.01 – 0.03 | 0.218 | -0.04 | -0.07 – -0.02 | **0.001** | 0.00 | -0.02 – 0.02 | 0.833 |
| TuG Seconds | 0.12 | -0.01 – 0.26 | 0.076 | 0.00 | -0.01 – 0.01 | 0.698 | 0.01 | -0.00 – 0.03 | 0.092 | 0.01 | -0.00 – 0.02 | 0.067 |
| BFI [Openness] | 1.67 | -0.63 – 3.97 | 0.153 | 0.06 | -0.13 – 0.25 | 0.521 | 0.02 | -0.22 – 0.26 | 0.862 | 0.12 | -0.05 – 0.30 | 0.171 |
| BFI [Agreeableness] | 1.45 | -1.30 – 4.20 | 0.299 | 0.09 | -0.14 – 0.32 | 0.438 | -0.06 | -0.35 – 0.23 | 0.696 | 0.11 | -0.10 – 0.33 | 0.284 |
| BFI [Conscientiousness] | 1.32 | -0.66 – 3.31 | 0.191 | 0.03 | -0.14 – 0.19 | 0.759 | -0.01 | -0.22 – 0.20 | 0.947 | 0.12 | -0.03 – 0.27 | 0.119 |
| BFI [Extraversion] | 0.28 | -1.99 – 2.54 | 0.812 | -0.03 | -0.21 – 0.16 | 0.787 | -0.02 | -0.26 – 0.21 | 0.839 | 0.03 | -0.14 – 0.20 | 0.738 |
| HCCQ | -0.74 | -1.28 – -0.20 | **0.007** | -0.06 | -0.10 – -0.01 | **0.014** | -0.03 | -0.08 – 0.03 | 0.354 | -0.02 | -0.06 – 0.02 | 0.283 |
| Number of Drugs | 0.07 | -0.10 – 0.23 | 0.430 | 0.01 | -0.01 – 0.02 | 0.315 | 0.04 | 0.02 – 0.06 | **<0.001** | -0.01 | -0.03 – -0.00 | 0.023 |
| Diagnosis [Cerebrovasc.] | -0.87 | -2.54 – 0.80 | 0.308 | -0.07 | -0.21 – 0.07 | 0.314 | -0.01 | -0.19 – 0.16 | 0.881 | -0.02 | -0.15 – 0.11 | 0.792 |
| Diagnosis [Neuromusc.] | -0.55 | -2.16 – 1.06 | 0.505 | -0.13 | -0.27 – -0.00 | **0.050** | -0.08 | -0.25 – 0.09 | 0.334 | 0.09 | -0.03 – 0.21 | 0.145 |
| Diagnosis [Epilepsy] | -0.55 | -3.54 – 2.45 | 0.720 | -0.09 | -0.34 – 0.16 | 0.498 | 0.11 | -0.21 – 0.43 | 0.492 | -0.12 | -0.35 – 0.11 | 0.319 |
| Diagnosis [Misc.] | -0.67 | -2.37 – 1.03 | 0.438 | -0.08 | -0.22 – 0.06 | 0.243 | -0.02 | -0.20 – 0.16 | 0.791 | 0.03 | -0.10 – 0.16 | 0.631 |
| SRH [1 - Excellent] | -15.68 | -23.28 – -8.09 | **<0.001** | 0.22 | -0.42 – 0.85 | 0.504 | -0.24 | -1.04 – 0.56 | 0.556 | -1.73 | -2.31 – -1.15 | **<0.001** |
| SRH [2 - Very good] | 9.65 | 3.26 – 16.03 | **0.003** | -0.19 | -0.72 – 0.34 | 0.484 | -0.00 | -0.68 – 0.67 | 0.998 | 1.27 | 0.78 – 1.75 | **<0.001** |
| SRH [3 - Good] | -5.88 | -10.32 – -1.45 | **0.009** | 0.11 | -0.25 – 0.48 | 0.544 | -0.10 | -0.57 – 0.37 | 0.681 | -0.76 | -1.10 – -0.42 | **<0.001** |
| SRH [4 - Poor] | 1.00 | -1.39 – 3.38 | 0.412 | -0.07 | -0.27 – 0.13 | 0.502 | 0.04 | -0.22 – 0.29 | 0.778 | 0.19 | 0.01 – 0.38 | **0.038** |
| Model Fit | N = 410, R^2^/R^2^ adjusted = 0.251 / 0.206, F(23, 386) = 5.624, p < .001 | | | N = 410, R^2^/R^2^ adjusted 0.121 / 0.068, F(23, 386) = 2.30, p < .001 | | | N = 410, R^2^/R^2^ adjusted 0.223 / 0.177, F(23, 386) = 4.815, p < .001 | | | N = 410, R^2^/R^2^ adjusted = 0.237 / 0.191, F(23,386) = 5. 202, p < .001 | | |
| Note: BDI = Beck Depression Inventory II, BFI = Big Five Inventory, MoCA = Montreal Cognitive Assessment, HCCQ = Healthcare Climate, Questionnaire, SAMS = Stendal Adherence to Medication Score, SRH = Self-Rated Health according to SF-36 Item 1, TuG = Timed Up and Go | | | | | | | | | | | | |

## **Supplement Table 2.** Linear Regression after Elastic Net Regularization for SAMS sub-scales using BDI items as predictors

| **Variable** | **Est.** | **CI** | **p** | **Est.** | **CI** | **p** | **Est.** | **CI** | **p** |
| --- | --- | --- | --- | --- | --- | --- | --- | --- | --- |
| Outcome | Model 1: SAMS: Forgetting | | | Model 2: SAMS: Missing Knowledge | | | Model 3: SAMS: *Modification* | | |
| Intercept | 0.28 | 0.21 – 0.34 | <0.001 | 0.38 | 0.28 – 0.49 | **<0.001** | 0.06 | -0.00 – 0.13 | 0.063 |
| BDI 1 |  | | |  | | | 0.03 | -0.05 – 0.10 | 0.465 |
| BDI 3 | 0.04 | -0.05 – 0.13 | 0.357 | 0.09 | -0.03 – 0.21 | 0.127 |  | | |
| BDI 4 |  | | | -0.08 | -0.18 – 0.02 | 0.101 | 0.07 | -0.03 – 0.17 | 0.144 |
| BDI 5 |  |  |  |  | | |  | | |
| BDI 9 | 0.07 | -0.05 – 0.18 | 0.263 |  |  |  |  |  |  |
| BDI 10 |  | | | 0.04 | -0.05 – 0.13 | 0.353 |  |  |  |
| BDI 11 | 0.02 | -0.05 – 0.08 | 0.640 |  |  |  | 0.03 | -0.03 – 0.09 | 0.305 |
| BDI 12 | 0.09 | 0.01 – 0.17 | **0.029** | 0.14 | 0.03 – 0.25 | **0.013** | 0.07 | -0.00 – 0.14 | **0.056** |
| BDI 14 | 0.04 | -0.05 – 0.13 | 0.417 | 0.07 | -0.06 – 0.20 | 0.283 |  | | |
| BDI 15 |  | | | -0.13 | -0.23 – -0.02 | **0.018** |  |  |  |
| BDI 16 |  |  |  |  | | | 0.03 | -0.01 – 0.07 | 0.158 |
| BDI 17 | 0.02 | -0.07 – 0.10 | 0.699 |  |  |  | 0.04 | -0.04 – 0.12 | 0.302 |
| BDI 18 |  | | | 0.04 | -0.04 – 0.13 | 0.330 |  | | |
| BDI 19 | 0.03 | -0.04 – 0.10 | 0.375 | 0.16 | 0.06 – 0.25 | **0.001** | 0.02 | -0.04 – 0.08 | 0.561 |
| BDI 20 | 0.06 | -0.01 – 0.13 | 0.093 |  | | | 0.05 | -0.02 – 0.11 | 0.144 |
| Model Fit | N = 730, R^2^ / R^2^ adjusted = 0.058 / 0.047  F(8, 721) = 5.509, p < .001 | | | N = 730, R^2^ / R^2^ adjusted = 0.058 / 0.047  F(8, 721) = 5.54, p < .001 | | | N = 730, R^2^ / R^2^ adjusted = 0.058 / 0.047  F(8, 721) = 5.52, p < .001 | | |
| Note: BDI = Beck Depression Inventory II; CI = Confidence Interval; SAMS = Stendal Adherence to Medication Score. Grey fields indicate that the variable was excluded from the model during elastic net regularization | | | | | | | | | |

## **Supplement Figure 1.** Spearman Correlations for SAMS Forgetting and BDI Items


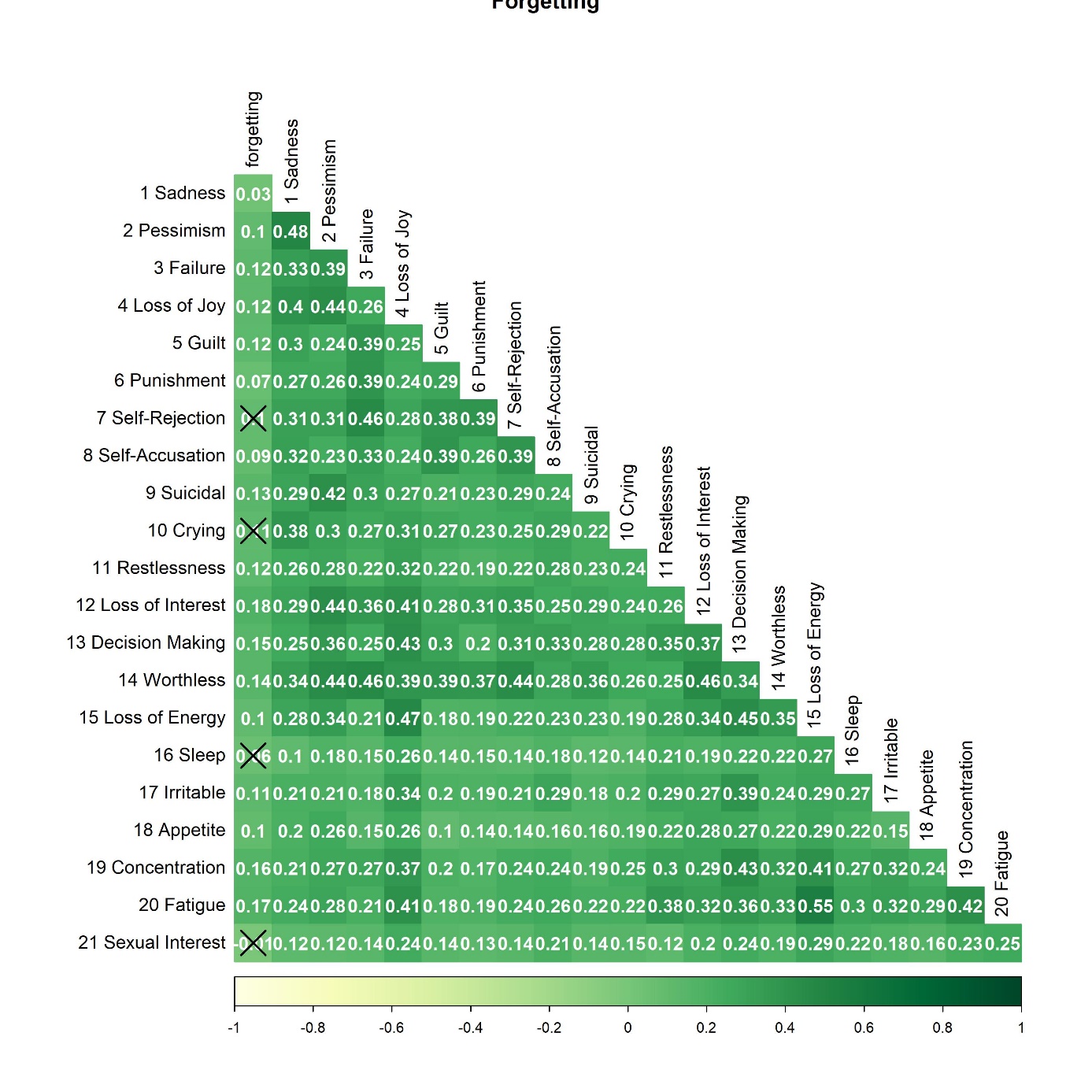


Note: BDI = Beck Depression Inventory II; SAMS = Stendal Adherence to Medication Score

## **Supplement Figure 2.** Spearman Correlations for SAMS Missing Knowledge and BDI Items


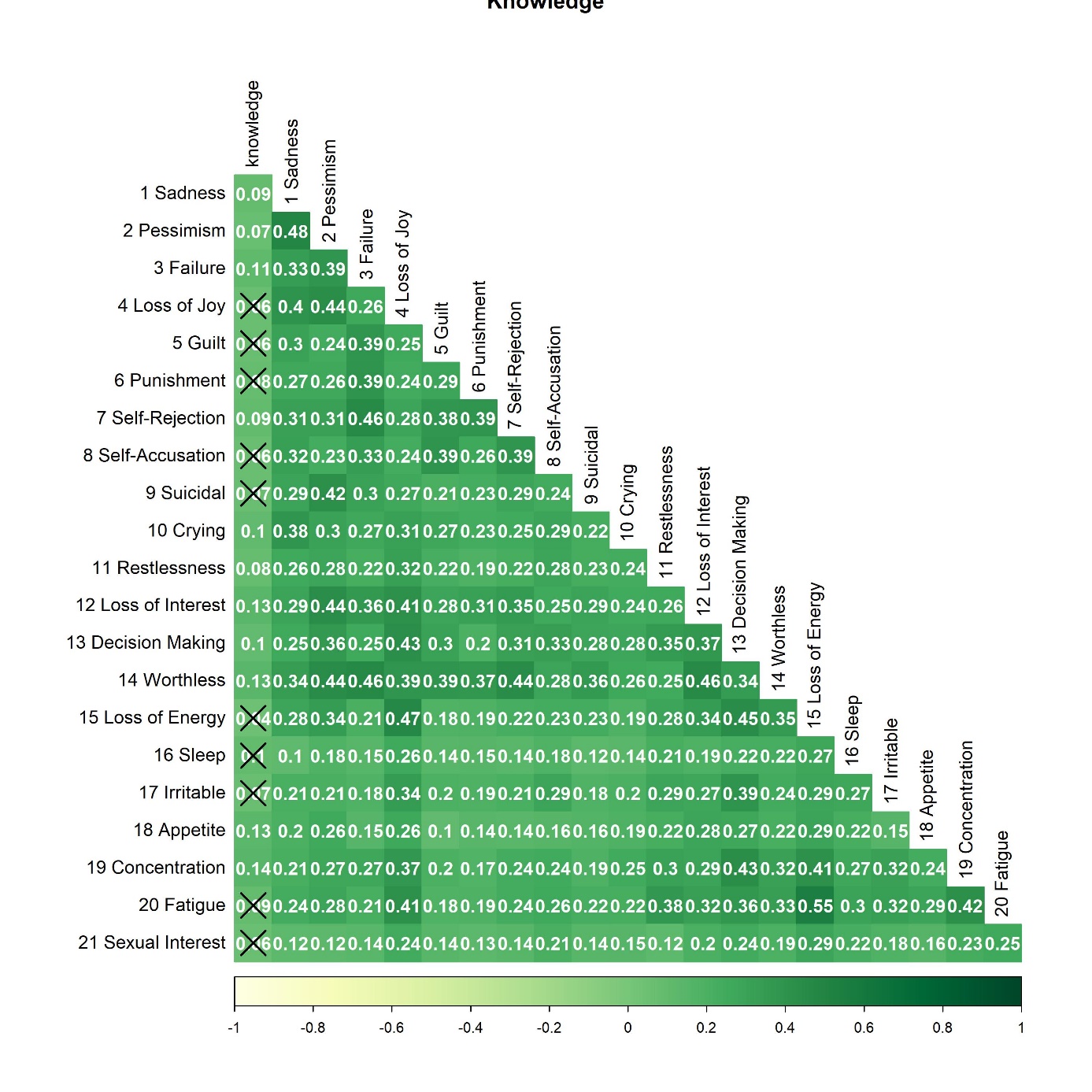


Note: BDI = Beck Depression Inventory II; SAMS = Stendal Adherence to Medication Score

## **Supplement Figure 3.** Spearman Correlations for SAMS Modification and BDI Items


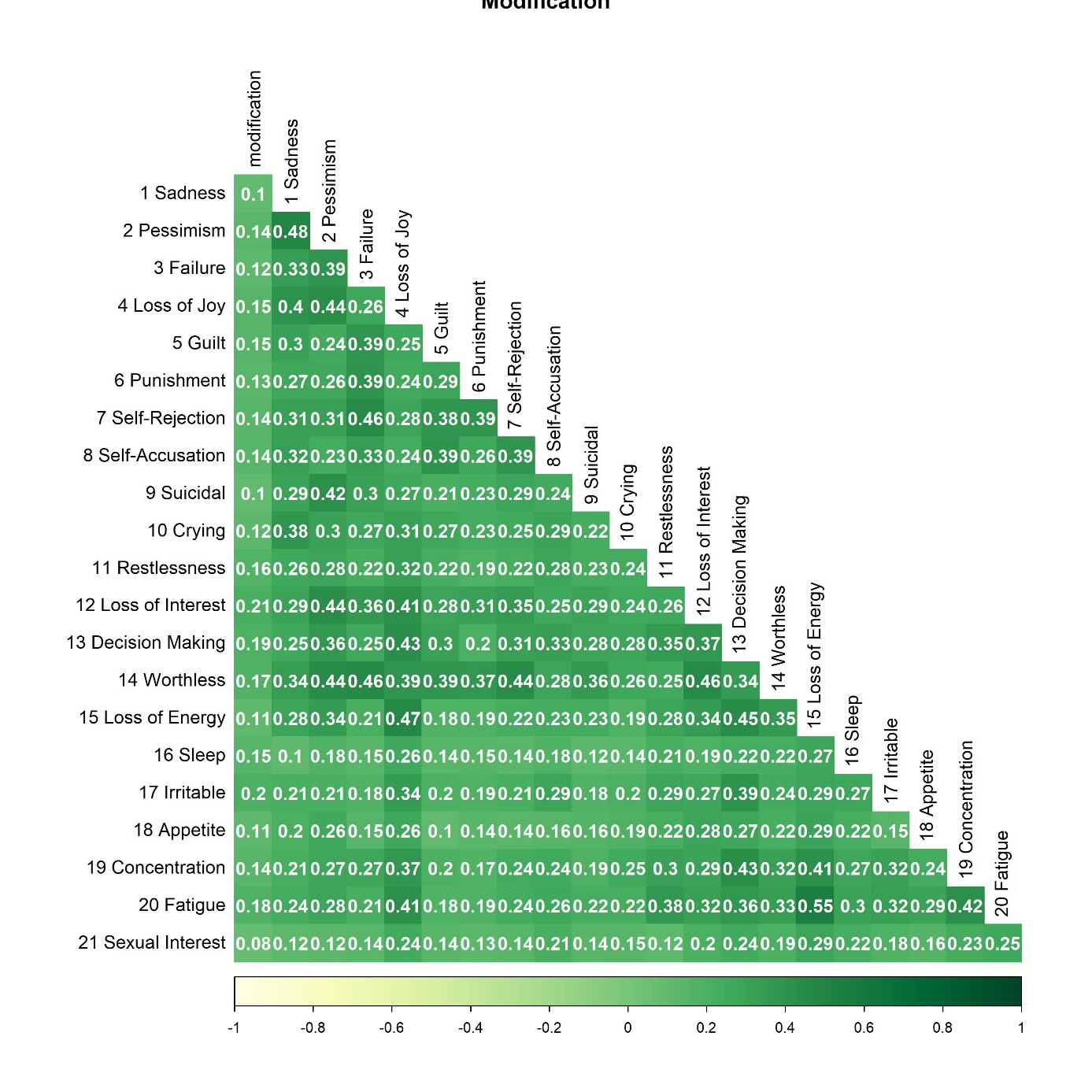


Note: BDI = Beck Depression Inventory II; SAMS = Stendal Adherence to Medication Score

## **Supplement Figure 4.** Network for SAMS Forgetting and BDI Items


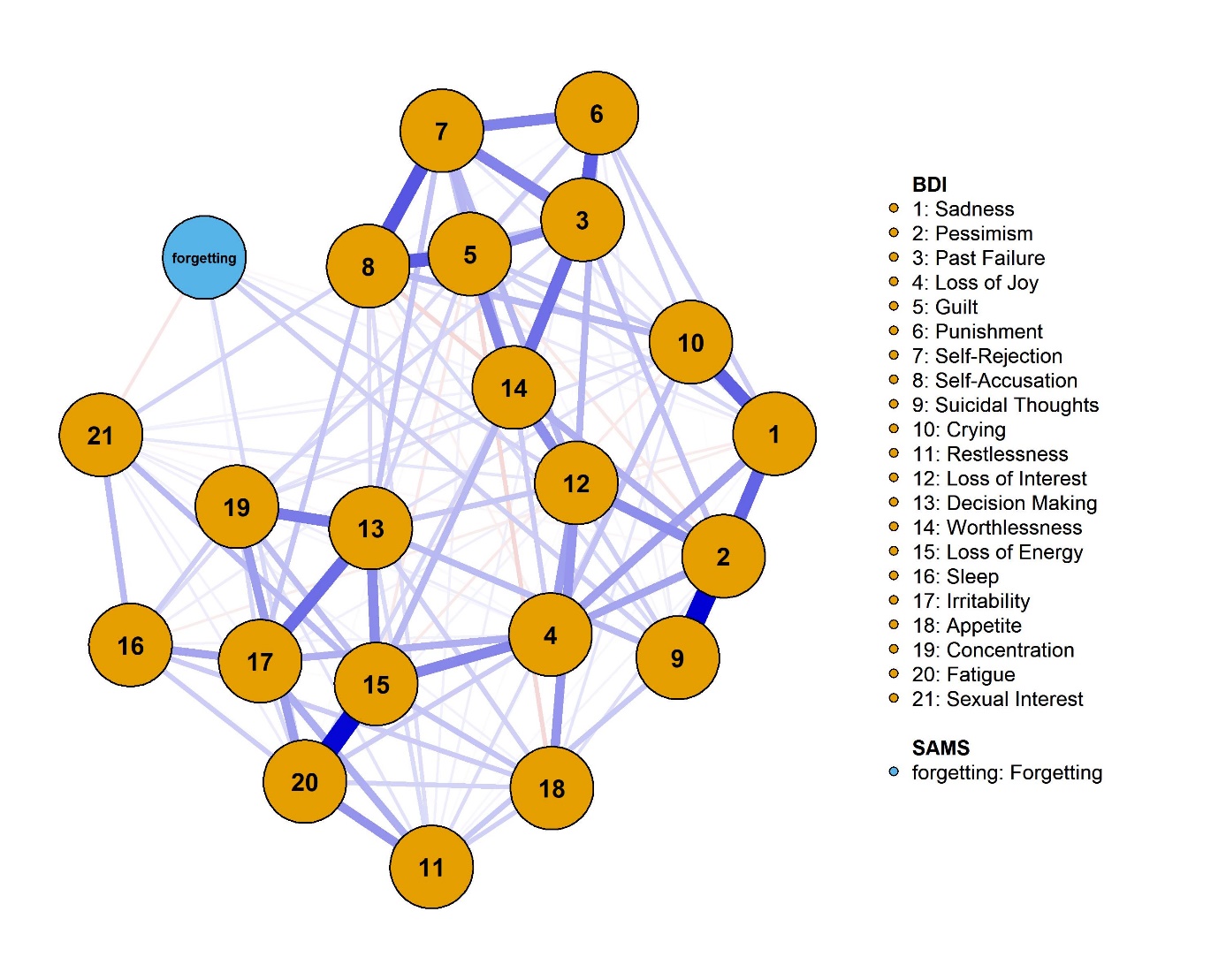


Note: CS-C = .595., displaying 133/231 edges. BDI = Beck Depression Inventory II; SAMS = Stendal Adherence to Medication Score

## **Supplement Figure 5.** Network for SAMS Missing Knowledge and BDI Items


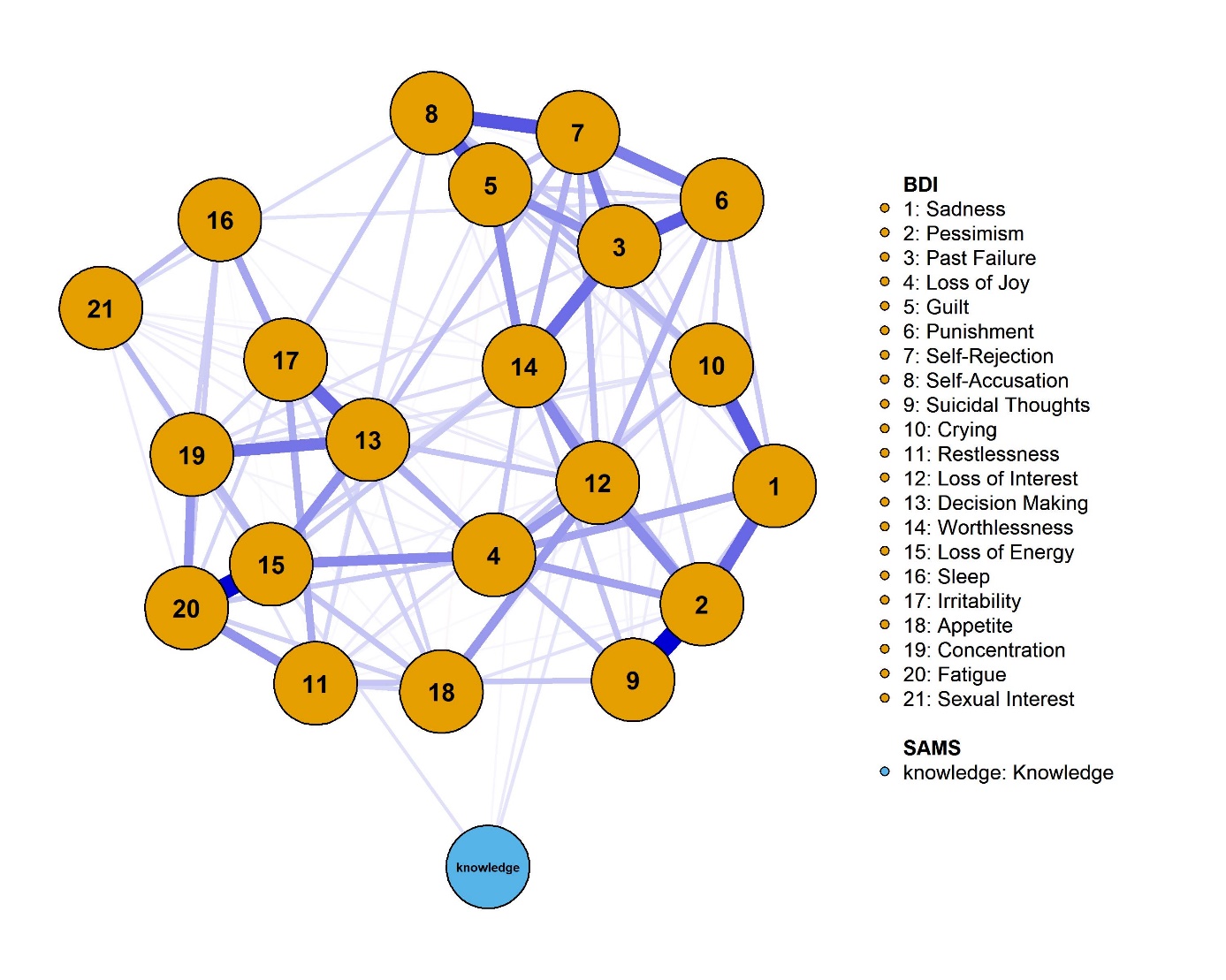


Note: CS-C = .595., displaying 116/231 edges. BDI = Beck Depression Inventory II; SAMS = Stendal Adherence to Medication Score

## **Supplement Figure 6.** Network for SAMS Modification and BDI Items


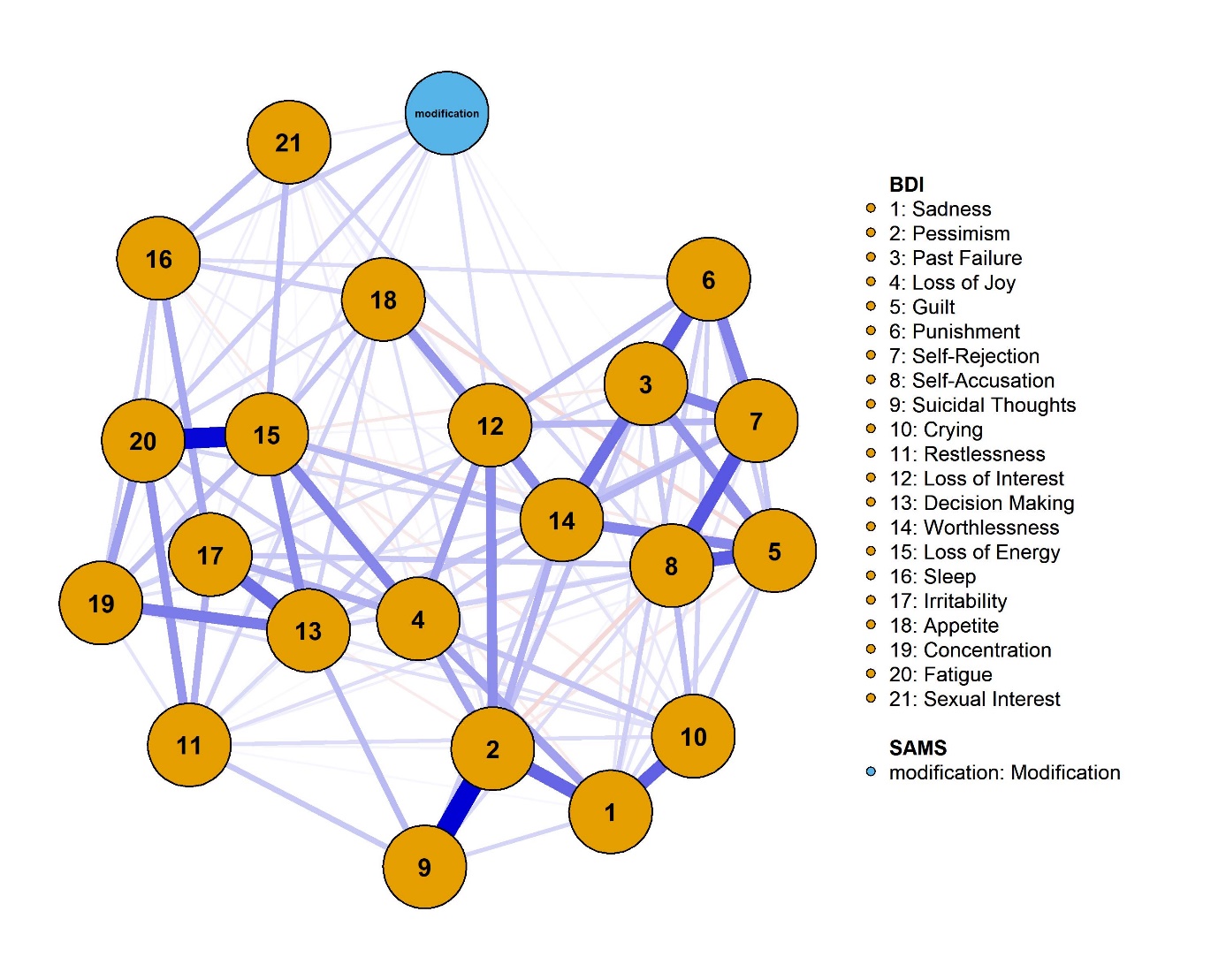


Note: CS-C = .516., displaying 136/231 edges. BDI = Beck Depression Inventory II; SAMS = Stendal Adherence to Medication Score
